# Structural deformation control in bridge construction based on error analysis and correction

**DOI:** 10.1371/journal.pone.0319844

**Published:** 2025-04-14

**Authors:** Juan Li, Rengye Zhao, Shengliang Cao

**Affiliations:** 1 The First Engineering Co., Ltd. of Shanxi Road & Bridge Construction Group, TaiYuan 030006, China; 2 CCCC First Highway Consultants Co., Ltd. Xi’an 710075, China; University of Zanjan, IRAN, ISLAMIC REPUBLIC OF

## Abstract

Structural deformation control of constructed bridges not only affects the alignment of the bridge, it is also the key to ensure safety. Factors such as temperature and time interval in actual construction can make the bridge deviate from the design state, therefore, this paper proposes a method based on error analysis and correction to eliminate these errors, and realize the structural deformation control in bridge construction control. The cantilever deflection in main girder is modeled and the effect of subsequent cantilevers on deflection at current section is further considered. The error in elevation caused by factors such as temperature and time interval is calculated, and a linear minimum variance estimate is employed to reduce this error. Practical engineering verification is carried out on a bridge in Shanxi, where the proposed error analysis method is further implemented by measuring the current cantilever elevation and comparing it with the original design value, with a purpose of obtaining a reasonable elevation for the next cantilever. The results show that, with the application of error analysis and correction, the elevation error generated during construction process is less than 20 mm, and the elevation error after the completion of bridge is less than 30 mm, the linear shape and internal condition of the bridge structure can also be further conformed to design requirements.

## Introduction

For the rapidly developing transportation industry, bridges have not only made access easier, which have also been extended to cross mountain streams, traverse bad geology, or meet other transportation needs [1,2]. The monitoring of bridge construction process is a systematic project, which consists of monitoring and control. Monitoring [3] refers to the use of data acquisition systems to measure large amounts of data during construction, such as stresses and deformations. The control of bridge construction [4] involves analyzing these data to guide the parameters of the next phase of construction.

There are a large number of studies on bridge monitoring. Soyoz et al. [5] used accelerometers to monitor vibration and structural stiffness changes in a new concrete bridge over a 5-year period, and stated that the study is significant for long-term bridge health assessment. Casas et al. [6] discussed the application of fiber optic sensors in bridge construction monitoring, and they pointed out that fiber optic sensors have the advantages of strong anti-interference ability and high accuracy when monitoring parameters such as bridge strain, temperature, and loading. Ntotsios et al. [7] utilized vibration sensors to capture bridge vibration values and further developed a bridge health monitoring system based on a Bayesian inference framework, and this approach was also validated by experiments. Farhangdoust et al. [8] summarized the application of NDT for health monitoring of closed joints in bridge construction, with different inspection methods for different types of closed joints. Similarly, Lin et al. [9] indicated the feasibility of Fiber Bragg Grating (FBG) sensors for bridge construction monitoring applications, which can not only monitor shrinkage and creep in bridge construction with high accuracy, it can also examine the prestress distribution in overall construction. Butler et al. [10] discuss the two main challenges of implementing fiber optic sensors in bridge construction, temperature compensation and strain variation, and they suggest the feasibility of using cable encapsulation and deploying glass fibers for sensors. As mentioned above, embedding sensors in bridge structures to capture changes in parameters such as temperature, stress, and strain is a key means of bridge inspection, which not only evaluates the health status of the bridge after completion, it also guides the operation of bridge construction process [11,12]. Orcesi et al. [13] effectively incorporate structural health monitoring (SHM) data into bridge structural assessment and prediction models, and its helps in bridge maintenance and operation. Gatti [14] noted that dynamic load tests can be used as a supplement to static load tests for bridge monitoring. Guzman-Acevedo et al. [15] pointed out the application of smart sensors for bridge monitoring, real project‘s results showed that fusion smart sensors are suitable for SHM of real-scale bridges. Gaxiola-Camacho et al. [16] proposed a method for bridge condition monitoring using GPS, which is realized by GPS reception of dynamic bridge displacements. He et al. [3] discussed the current challenges and future research directions for integrated SHM systems with respect to the characteristics of bridges.

Although the placement of various sensors can effectively obtain bridge parameters, further parameter processing to obtain bridge health status and bridge construction control is particularly critical. Liu et al. [17] proposed a method for evaluating the performance of bridge systems based on structural health monitoring, with strain data from a long-term inspection of a bridge in US. Li et al. [18] analyzed the stress and deformation data obtained from bridge monitoring, then proposed an analytical method for traffic load effects and expansion joint temperatures, with final proposals for bridge service performance assessment and service life prediction. Jang et al. [19] established a structural health monitoring system based on wireless smart sensor network (WSSN) and combined it with finite element modeling to evaluate the bridge condition. He et al. [3] analyzed the data signals captured by sensors buried in bridge structures with artificial intelligence-based data processing methods, and developed a bridge structural health monitoring system. It can be seen that the implementation of monitoring data processing and analysis for completed bridges is the primary measure to maintain the health of bridges. Farahmand et al. [20] developed the optimal parameters for improving the support system in cable arch bridges to suppress force fluctuations and overstressing problems.

Further, since the bridge construction is phased, the stresses and deformations of its structure in each phase can be predicted by modeling and actual measurements [21–23], and once it is found that there is a large error between the actual values monitored and calculated predicted values during construction, it is necessary to correct the relevant calculations in construction, which is the significance of bridge construction control. To assure bridge quality and construction safety, Cho et al. [24] introduced a finite element analysis method by using the obtained data from sensors, to quantitatively assess risk during the bridge construction phase. Wu et al. [25] used simulation to generate a bridge construction schedule, while it took into account the available resources and the interdependence of individual tasks, it was unable to finely examine the effects of structural forces and settlement on the construction schedule after this phase. Tan et al. [26] conducted a study of prestressed concrete bridges under construction, where forward and backward analyses were used to calculate structural deformations, respectively, and the pre-tilt angle of each cantilevered construction pipe section was obtained, which serves as a basis for construction control. Qin et al. [27] described the application of stress-free state theory to bridge construction control and noted that the theory can guide staged construction. There have also been studies utilizing machine learning as a basis for bridge construction control [28–30].

Reviewing the aforementioned literature, various approaches such as machine learning, finite element analysis, and simulation techniques have been introduced to implement bridge construction control. However, the above-mentioned methods can produce large errors in practical application owing to the influence of various factors such as construction environment, operation mode and human intervention. For example, complex soil composition, rock types and even climatic conditions will greatly affect the finite element analysis, while the maintenance management of bridges during construction is difficult to realize in all kinds of prediction models, and what’s more, the human errors occurring during construction are even more uncontrollable. Therefore, this paper proposes a structural deformation control of bridge based on parameter identification and error analysis. The deflection calculation of each cantilever section is first introduced based on the conjugate beam method, which is the basis for obtaining the actual elevation. Then the errors brought about by temperature, time and process changes are modeled. Finally, the theoretical value of the cantilever elevation during bridge construction was calculated. When the theoretical value of the elevation has a large error with the measured value, the error model is calibrated so that the predicted value converges to the measured value. The method will be validated in real engineering applications.

## Model for structural deformation control of bridge

**Fig 1:**
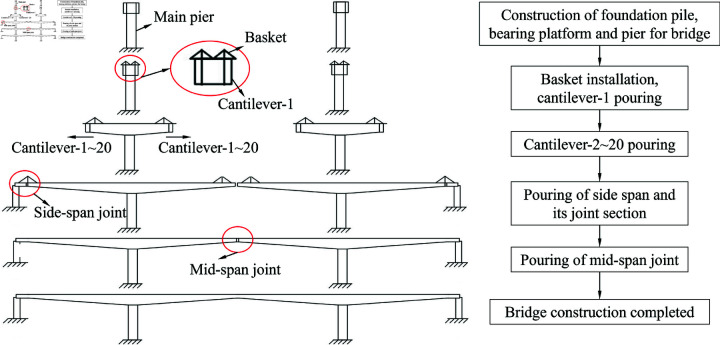
Schematic diagram and process of construction for a bridge in Shanxi Province.

The study was exempt from institutional ethics committee approval. Regardless of construction method, the bridge structure in the construction process will always produce deformation, and the deformation will be affected by many factors, which will easily make the actual position of constructed bridge (elevation, planar position) deviates from the expected state. Thus, it is difficult for the bridge to be joined together smoothly, or the permanent linearity of bridge is not in conformity with design requirements. Taking the bridge construction in Shanxi Province as an example, the bridge has a total length of 681 m, with a width of 15 m for each left and right span its engineering phases are shown in Fig. 1. And which can be roughly divided into six stages, including pier construction, each cantilever pouring, side-span joining and main-span joining. Specifically, the cantilever sections were poured in stages and the completed cantilever was fixed at one end and free at the other. Therefore, the cantilever is prone to settlement and deformation due to temperature and workmanship factors, which deviate from the required condition.

**Fig 2:**
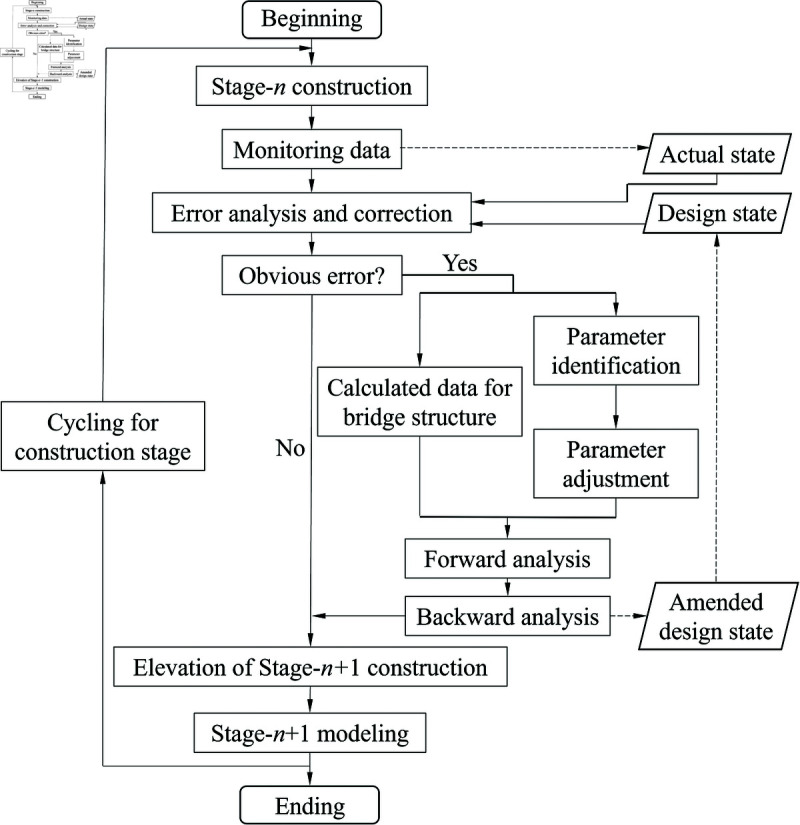
The construction control process of bridge deformation based on parameter identification and error analysis.

Construction control serves to ensure the safety of the structure during construction and guarantee that the linear and internal state of the structure conforms to the design requirements. However, as construction control is inevitably subject to errors due to, for example, temperature, time and process variations. Therefore, in order to predict the reasonable mold elevation of the next cantilever, it is necessary to observe the deflection changes of the main beams in each construction process of the current cantilever to obtain the measured state. The control parameter error analysis and modified prediction control method is used to compare and analyze the measured state with the original ideal state, to filter out the random error and make a judgment on the systematic error. If there is an obvious systematic error, the first requirement is to correct the data related to the structural calculation, and the parameter error identification is adjusted, and then forward and backward analysis is carried out. The construction control process of bridge deformation based on parameter identification and error analysis is shown in Fig. 2.

### Cantilever deflection

In the theoretical analysis, the deflection calculation of each cantilever section is first introduced based on the conjugate beam method, which is the basis for obtaining the actual elevation. Then the errors brought about by temperature, time and process changes are modeled. Finally, the theoretical value of the cantilever elevation during bridge construction was calculated. When the theoretical value of the elevation has a large error with the measured value, the error model is calibrated so that the predicted value converges to the measured value.

To implement structural deformation in bridge construction control and further obtain the construction elevation of next cantilever section, the elevation of currently constructed cantilever is required to be measured first. The measured elevation contains the pier elevation and main girder deflection, by calculating their theoretical values and further comparing them with the measured values, the error can be checked and the next step of error analysis can be facilitated.

**Fig 3:**
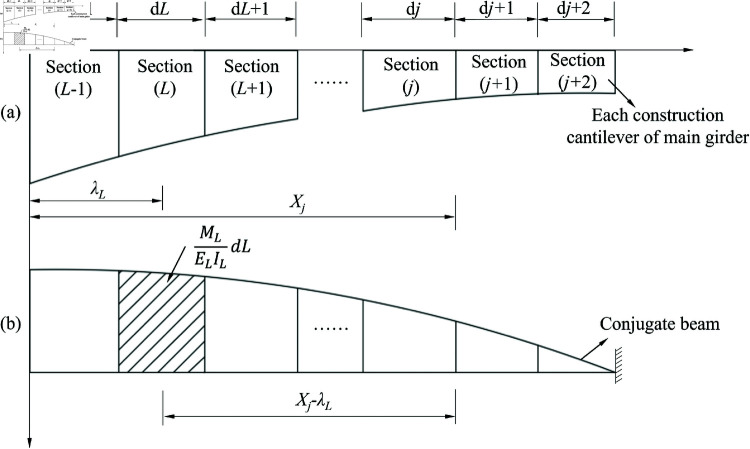
Calculation of bridge deflection under conjugate been method. (**a**) Structural and (**b**) cantilever deflection calculation diagram for the bridge construction.


For the cantilever deflection calculation of varied sections, the conjugate beam method is employed to solve the problem [31], as shown in Fig. 3. The conjugate beam method exhibits extremely high accuracy in bridge engineering, especially in the fitting of deflection curves of simply supported Euler-Bernoulli beams. In the mid-span region where the deflection is large, the relative error of fitting can even be reduced to about 0.5*%*. This means that the conjugate beam method can predict the deflection changes of bridges very accurately and provide reliable data support for bridge design and monitoring. It is because of the high accuracy and convenient calculation of the conjugate beam method that it is widely used in bridge engineering. When section *j* is constructed, the deflection at section, where any point *K* on the main beam is located, can be calculated as [32]

$$\begin{array}{ccc} \begin{array}{c} Y(Xc)=\overset{k}{\underset{L=1}{\sum }}\frac{M(\lambda )}{E_{L}I_{L}}\left(X_{k}-\lambda_{L}\right)dL=\overset{k}{\underset{L=1}{\sum }}R_{KL}∙M(\lambda )\\ R_{KL}=\frac{X_{k}-\lambda_{L}}{E_{L}I_{L}}dL \end{array} & & \end{array}$$ (1)

where $X_{k}$ and $\lambda_{L}$ represent the transverse coordinates of observation point *K* and the midpoint of section *L*, respectively. $E_{L}$, $I_{L}$ and $d_{L}$ are the modulus of elasticity, inertia moment and length of section *L*, respectively. Specifically, $M(\lambda_{L})$ denotes the bending moment experienced at the midpoint of section *L* after section *j* has been built, which is formed by the weight and applied tensile force of section *L* and subsequent sections built thereafter, and it can be calculated as


$$\begin{array}{ccc} M(\lambda_{L})=\overset{j-1}{\underset{i=L}{\sum }}P_{Li}e_{L}l(t-{t^{\prime }}_{i})-\overset{j}{\underset{i=L}{\sum }}Q_{i}L_{Li}l(t-t_{i}) & & \end{array}$$ (2)

The tension force that forms the moment acted on section *L* refers to the hanging basket cable tension, e.g., the tension force of cable *i* on section *L* is denoted by $P_{Li}$. $Q_{i}$ means the self-weight of beam in section *i*, and $L_{Li}$ is the equivalent force arm from section *i* to the center of section *L*. Additionally, the step function $l(t\;-\;t_{i})$ represents the moment of elastic deformation.

On the other hand, the pier height is compressed by the beam and self-weight, which also causes the change in cantilever elevation. After section *j* is built, the height of point *K* on girder can be expressed as


$$\begin{array}{ccc} H_{kj}=\overset{k}{\underset{L=1}{\sum }}R_{kL}\left[\overset{j-1}{\underset{i=L}{\sum }}e_{L}P_{Li}l(t-{t^{\prime }}_{i})-\overset{j}{\underset{i=L}{\sum }}Q_{i}L_{Li}l(t-t_{i})\right]+H_{0}-\frac{2H_{0}}{EB_{0}}\overset{j}{\underset{i=0}{\sum }}Q_{i}(t-t_{i}) & & \end{array}$$ (3)

where $t_{i}$ and $t_{i}^{\prime }$ represent the moment of completion and tensioning of section *i*, respectively, and $Q_{0}$ denotes one quarter of the pier’s self-weight. *E* and $B_{0}$ are the elastic modulus and cross-sectional area of the pier, respectively, and $H_{0}$ shows the relative height.

After tensioning the prestress in section *j*, the height of point *K* can be denoted as


$$\begin{array}{ccc} H_{{}_{kj}}^{\phi }=\overset{k}{\underset{L=1}{\sum }}R_{kL}\left[\overset{j}{\underset{i=L}{\sum }}P_{Li}e_{L}l(t-{t^{\prime }}_{i})-\overset{j}{\underset{i=L}{\sum }}Q_{i}L_{Li}l(t-t_{i})\right]+H_{0}-\frac{2H_{0}}{EB_{0}}\overset{j}{\underset{i=0}{\sum }}Q_{i}l(t-t_{i}) & & \end{array}$$ (4)

Combining Eqs. 3 and 4, the difference in elevation at point *K* before and after tensioning the prestress for section *j* can be obtained, and which can be expressed as


$$\begin{array}{ccc} Y_{KL}^{\phi }\approx H_{Kj}^{\phi }-H_{Kj}^{0}=\overset{k}{\underset{L=1}{\sum }}R_{kL}P_{Lj}e_{L}l(t-{t^{\prime }}_{j}) & & \end{array}$$ (5)

Furthermore, the difference in elevation at point *K* after the section *j* + 1 is poured can be calculated as


$$\begin{array}{ccc} Y_{Kj+1}^{0}=H_{Kj+1}^{0}-H_{Kj}^{\phi }=-\overset{k}{\underset{L=1}{\sum }}R_{kL}L_{Lj+1}l(t-t_{j+1})-\frac{H_{0}}{E_{0}B_{0}}Q_{j+1}l(t-t_{j+1}) & & \end{array}$$ (6)

### Error analysis

The above theoretical calculation of elevation only considers the cantilever’s elastic deformation. However, climate change, construction period, fluctuation of material properties and construction technology, etc. will all lead to the difference between the data used in design and actual construction, and these differences are the main reasons for error between measurement and design [33]. By analyzing the influence of each factor and establishing the functional relationship between these differences and measurement error, accurate bridge construction control can be achieved [34]. The effect of pier height on deflection is analyzed firstly. Since the piers are high, temperature changes affect the measured values; moreover, the piers carry their own weight and the weight of beams added section by section, and their load and compression deformation are increasing. At the same time, the creep deformation and elastic deformation increases proportionally, the proportion coefficient is related to loading time [35], which is expressed as


$$\begin{array}{ccc} (t,\tau )=\{\begin{array}{c} 0\begin{array}{cccc} & & & \begin{array}{ccc} & & \;\;\;t<\tau \end{array} \end{array}\\ \psi_{k}(k+(1-k)e^{-v\tau })(1-e^{-v(t-\tau )})\;t\geq \tau \end{array} & & \end{array}$$ (7)

where *t* is the time from completion of each cantilever, and *τ* represents the loading time. Since the cantilever beams were built at different times for each section, the starting point for loading time calculation was different. Therefore, the loading time is given by $\tau =t_{L}-t_{i}$ to facilitate the subsequent calculation, where $t_{L}$ and $t_{i}$ denote the build and load moments of section *L*, respectively. Thus, the pier height in subjected to continuous compressive deformation can be expressed as


$$\begin{array}{ccc} \begin{array}{c} \varsigma_{j}(t,T)=H_{0}\left\{1+\alpha \Delta T-\frac{2}{EB_{0}}\overset{j}{\underset{i=0}{\sum }}\left[1+\psi (t,t_{i,}t_{0})\right]Q_{i}\right\}\\ \left(t,t_{i},t_{0})=\psi_{k}(k+(1-k)e^{-v(t_{i}-t_{0})})(1-e^{-v(t-t_{i})}\right) \end{array} & & \end{array}$$ (8)

where $t_{0}$ indicates the moment of pier completion.

Next, the beam deflection analysis is performed. The beam deflection is formed by self-weight and cable tension [36]. The effect of self-weight on the deflection is continuous, and its creep law is


$$\begin{array}{ccc} \beta_{Kj}(t)=-\overset{k}{\underset{L=1}{\sum }}R_{kL}\overset{j}{\underset{i=L}{\sum }}Q_{i}l_{iL}\left[1+\psi (t,t_{i},t_{L})\right] & & \end{array}$$ (9)

The creep pattern of tension deflection caused by steel cables is different from that of self-weight, which is caused by that the steel cables will be loosened under the effect of creep, and the tensile force has to be decreased. Therefore, the creep process of tension deflection can be given as


$$\begin{array}{ccc} \eta_{kj}(t)=\overset{k}{\underset{L=1}{\sum }}R_{kL}e_{L}\overset{j}{\underset{i=L}{\sum }}P_{Li}e^{-\psi } & & \end{array}$$ (10)

Combining Eqs. 8, 9 and 10, the difference in elevation at point *K* before and after tensioning of section *j* can be obtained and it is expressed as


$$\begin{array}{ccc} \xi_{kj}^{\phi }=(\eta_{kj}^{\left({t^{\prime }}_{j}\right)}+\beta_{kj}^{\left({t^{\prime }}_{j}\right)}+\varsigma_{j}^{\left({t^{\prime }}_{j}{\tau^{\prime }}_{j}\right)})-(\eta_{k,j-1}^{\left(t_{j}\right)}+\beta_{k,j}^{\left(t_{j}\right)}+\varsigma_{kj}^{\left(t_{j}\tau_{j}\right)}) & & \end{array}$$ (11)

Eq. 11 indicates that any difference between the moment of measurement and ambient temperature will bring about a measurement error. The above equation can be expanded as


$$\begin{array}{ccc} \xi_{kj}^{\phi }=\Delta t\eta_{j-1}+\Delta t\beta_{j}+\Delta T\varsigma_{j}+\overset{k}{\underset{L=1}{\sum }}R_{kL}e_{L}P_{Li}e^{-\psi (t_{j},{t^{\prime }}_{j,}t_{L})} & & \end{array}$$ (12)

In particular, it is noticed that $-\psi (t_{j},t_{j,}^{\prime }t_{L})=0$ and the last term is formally the same as the calculated elevation difference shown in Eq. 5. However, considering the differences in process and construction materials, the elastic modulus of actual project will differ from the design data, and the last term in Eq. 12 can be corrected as


$$\begin{array}{ccc} \overset{k}{\underset{L=1}{\sum }}{R_{kL}P_{Lj}e_{L}=(\overset{k}{\underset{L=1}{\sum }}R_{kL}P_{Lj}e_{L})}_{Design}-(\overset{k}{\underset{L=1}{\sum }}R_{kL}P_{Lj}e_{L})\frac{\Delta E}{E} & & \end{array}$$ (13)

The other terms in Eq. 12 can be expanded separately and expressed as


$$\begin{array}{ccc} \begin{array}{c} \Delta t\varsigma_{j}=H_{0}\alpha \Delta T\\ \Delta T\varsigma_{j}=-\frac{2H_{0}}{EB_{0}}\overset{j}{\underset{i=0}{\sum }}Q_{i}f(t_{j},t_{i},t_{0})\Delta \psi_{\varsigma }\\ \Delta t\eta_{j-1}=\overset{k}{\underset{L=1}{\sum }}R_{kL}l_{L}\overset{j-1}{\underset{i=L}{\sum }}P_{Li}e^{-\psi (t_{j},{t^{\prime }}_{j},t_{L}})f(t_{j},{t^{\prime }}_{j},t_{L})\psi_{\eta }\\ \Delta t\beta_{j}=-\overset{k}{\underset{L=1}{\sum }}R_{kL}\overset{j}{\underset{i=L}{\sum }}Q_{i}l_{Li}f(t_{j},{t^{\prime }}_{j},t_{L})\psi_{\beta } \end{array} & & \end{array}$$ (14)

where $f(t_{1},t_{2},t_{3})=(k\;+\;(1\;-\;k)e^{-v(t_{2}-t_{3})})e^{-v(t_{1}-t_{2})}v\Delta t$, *α* is the temperature coefficient of pier, *Δt* and *ΔT* represent the time interval and temperature difference between the two measurements before and after tensioning, respectively. It is obvious from Eqs. 13 and 14 that the measured values contain not only the calculated values, there is also the error caused by ambient temperature, construction period and technological process. Further combining Eqs. 5 and 12, an analytical expression for the relationship between the measurement error and above factors can be obtained by


$$\begin{array}{ccc} \delta_{jk}^{\varphi }\approx \xi_{kj}^{\phi }-Y_{KL}^{\phi }=\mu_{0}^{\varphi }\alpha +\mu_{0}^{\varphi }\frac{\Delta E}{E_{0}}+\mu_{2}^{\varphi }\Psi_{\tau }+\mu_{3}^{\varphi }\frac{\Delta E}{E_{b}}+\mu_{4}\Psi_{\eta }+\mu_{5}\Psi_{\beta } & & \end{array}$$ (15)

where $\Delta E∕E_{0}$ and $\Delta E∕E_{b}$ are the errors in elastic modulus for piers and girders, respectively. $\Psi_{\tau }$, $\Psi_{\eta }$ and $\Psi_{\beta }$ represent the creep coefficients of deflection owing to pier, girder tensioning and girder self-weight, respectively. Additionally, $\mu_{0}^{\varphi }$ to $\mu_{5}^{\varphi }$ are detailed as follows


$$\begin{array}{ccc} \begin{array}{c} \mu_{1}^{\varphi }=0\\ \mu_{2}^{\varphi }=-\frac{2H}{E_{0}B_{0}}\overset{h}{\underset{j-0}{\sum }}Q_{i}f(t_{j},t_{i},t_{0})\\ \mu_{3}^{\varphi }=-\overset{k}{\underset{L=1}{\sum }}R_{kL}P_{Lj}e_{L}\\ \mu_{4}^{\varphi }=\overset{k}{\underset{L=1}{\sum }}R_{kL}e_{L}\overset{j-1}{\underset{i=L}{\sum }}P_{Li}e^{-\psi (t_{j},t_{i},t_{L})}\cdot f(t_{j},t_{i},t_{L})\\ \mu_{5}^{\varphi }=\overset{k}{\underset{L=1}{\sum }}R_{kL}\overset{j}{\underset{i=L}{\sum }}Q_{i}l_{Li}f(t_{j},t_{i},t_{L}) \end{array} & & \end{array}$$ (16)

Similarly, for the measurement error before and after the cantilever casting of section *j* + 1, it can be expressed as


$$\begin{array}{ccc} \begin{array}{c} \delta_{k,j+1}^{0}=\mu_{0}^{0}\alpha +\mu_{1}\frac{\Delta E}{E_{0}}+\mu_{2}^{0}\Psi_{\tau }+\mu_{3}^{0}\frac{\Delta E}{Eb}+\mu_{4}^{0}\Psi_{\eta }+\mu_{5}^{0}\Psi_{\beta }\\ \mu_{0}^{0}=H_{0}\Delta t\\ \mu_{1}^{0}=\frac{2H_{0}}{B_{0}E_{0}}Q_{j+1}\\ \mu_{2}^{0}=-\frac{2H_{0}}{B_{0}E_{0}}\overset{j}{\underset{i=0}{\sum }}Q_{i}f(t_{j},t_{i},t_{0})\\ \mu_{3}^{0}=-\overset{k}{\underset{L=1}{\sum }}R_{kL}\cdot l_{L,j+1}\cdot Q_{j+1}\\ \mu_{4}^{0}=-\overset{k}{\underset{L=1}{\sum }}R_{kL}e_{L}\overset{j}{\underset{i=L}{\sum }}P_{Li}e^{-\Psi (t_{j},t_{i},t_{L})}\cdot f(t_{j},t_{i},t_{L})\\ \mu_{5}^{0}=-\overset{k}{\underset{L=1}{\sum }}R_{kL}\overset{j}{\underset{i=L}{\sum }}Q_{i}l_{Li}\cdot f(t_{j},t_{i},t_{L}) \end{array} & & \end{array}$$ (17)

Based on the above deflection calculation and error analysis, it is possible to utilize the error expressions shown in Eqs. 16 and 17, and the errors between calculations and measurements can be obtained for all cantilever construction sections, and the relationships between these errors and temperature coefficients, elastic modulus, and creep coefficients can also be studied. It is worth noting that none of these parameters are known accurately, and it is necessary to utilize the measured data from construction to estimate them.

### Parameter estimation and deflection correction

The six parameters (*α*, $\Delta E∕E_{0}$, $\Delta E∕E_{b}$, $\Psi_{\tau }$, $\Psi_{\eta }$ and $\Psi_{\beta }$) in the error model derived above are the parameters which need to be estimated. Linear minimum variance is employed to perform these parameters [37,38]. In this estimation method, the variance of estimated parameters and the weighted average of variance for measurement noise are continuously utilized to obtain the evaluated values. Where the design data is used as the initial value *X*(0), the initial variance *P*(0) is the error range of field construction parameters, which can be given empirically. The variance for measurement noise *R*(*x*) is available from the data preprocessing. The linear minimum variance estimate can be expressed as


$$\begin{array}{ccc} \begin{array}{c} X(k)=X(k-1)+G(k)(y(k)-h(k)X(k-1))\\ G(k)=P(k-1)H^{\prime }(k)(H(k)P(k-1))H^{\prime }(k)+R(k))-1\\ P(k)=P(k-1)-G(k)H(k)P(k-1) \end{array} & & \end{array}$$ (18)

By solving sequentially and recursively for the parameters at the time of construction on each cantilever section, their valuation is obtained and calculated as


$$\begin{array}{ccc} \hat{X}(k)=(\alpha ,\Delta E∕Eo,\Psi_{\varsigma },\Delta E∕Eb,\Psi_{\eta },\Psi_{\beta }) & & \end{array}$$ (19)

Notably, after processing one measurement point, the measurement variance *R*(*x*) and the measurement value are scalars, and the inverse of the computation for gain matrix *G*(*x*) can be written as *Y* ( *k* ) = *ξ* ( *k* ) − *H* ( *k* ) *X* ( *k* ) ⋯ ⋯ *K* = 1 ∼ 100.

These valuations can be used to correct the calculation parameters; and the corrected parameters can be further realized to forecast the deflection or elevation of later cantilever construction, which is an effective measure to improve the construction accuracy.

### Structural deformation control in actual bridge construction control

Taking a bridge in Shanxi shown in Fig. 1 as an example, the whole construction process is regarded as a system, which is the noise-disturbed process of error analysis and correction for control coefficients with physical prototypes. Therefore, the gray system theory model [39] is selected to predict and control the bridge construction.

According to the requirements of gray system theory model, the number of control parameters should not be less than 4. Therefore, when the elevation data observation of cantilever construction in the bridge in Shanxi is carried out, the previous 4 sections of cantilever should be erected according to design state and construction knowledge. The elevation should be observed in the same time interval and the measured elevation of each cantilever should be corrected by the temperature effect, thus, a data series can be formed as follows


$$\begin{array}{ccc} H_{i}^{\left(0\right)}=(H_{i}^{\left(0\right)}(1),\;H_{i}^{\left(0\right)}(2),\;H_{i}^{\left(0\right)}(3),\;H_{i}^{\left(0\right)}(4)) & & \end{array}$$ (20)

where *i* = 1, 2, 3 and $H_{i}^{\left(0\right)}$ represents the original measured elevation data series. $H_{i}^{\left(0\right)}(1)$, $H_{i}^{\left(0\right)}(2)$, $H_{i}^{\left(0\right)}(3)$ and $H_{i}^{\left(0\right)}(4)$ denotes the measured cantilever elevations of sections 1, 2, 3 and 4, respectively.

The data series in Eq. 20 after accumulating (1-AGO) can be expressed as


$$\begin{array}{ccc} \begin{array}{c} H_{i}^{\left(1\right)}=(H_{i}^{\left(1\right)}(1),\;H_{i}^{\left(1\right)}(2),\;H_{i}^{\left(1\right)}(3),\;H_{i}^{\left(1\right)}(4))\\ H_{i}^{\left(1\right)}(1)=H_{i}^{\left(0\right)}(1)\;i=1,2,3\\ H_{i}^{\left(1\right)}(k+1)=H_{i}^{\left(0\right)}(k+1)+H_{i}^{\left(1\right)}(k)\;k=1,2,3 \end{array} & & \end{array}$$ (21)

where$H_{i}^{\left(1\right)}$ can be established as a differential equation in the following whitened form


$$\begin{array}{ccc} \frac{dH_{i}^{\left(1\right)}}{dt}+a_{i}H_{i}^{\left(1\right)}=b_{i} & & \end{array}$$ (22)

To simplify the solution of differential equation, the parameter column is set to


$${â}_{i}=\left[\begin{array}{c} a_{i}\\ b_{i} \end{array}\right].$$

The solution is further carried out by using the least squares method as follows


$$\begin{array}{ccc} \begin{array}{c} {â}_{i}={\left(B_{i}^{T}B_{i}\right)}^{-1}B_{i}^{T}Y_{iN}\\ B_{i}=\left[\begin{array}{c} -\frac{1}{2}(H_{i}^{\left(1\right)}(1)+H_{i}^{\left(1\right)}(2))1\\ -\frac{1}{2}(H_{i}^{\left(1\right)}(2)+H_{i}^{\left(1\right)}(3))1\\ -\frac{1}{2}(H_{i}^{\left(1\right)}(3)+H_{i}^{\left(1\right)}(4))1 \end{array}\right]\\ Y_{iN}=\left[H_{i}^{\left(0\right)}(2),H_{i}^{\left(0\right)}(3),H_{i}^{\left(0\right)}(4)\right] \end{array} & & \end{array}$$ (23)

The solution of differential equation in whitened form shown in Eq. 22 is finally obtained by


$$\begin{array}{ccc} {Ĥ}_{i}^{\left(1\right)}(k+1)=\left(H^{\left(0\right)}(1)-\frac{b_{i}}{a_{i}}\right)e^{-a_{i}k}+\frac{b_{i}}{a_{i}} & & \end{array}$$ (24)

The values of elevation change for each cantilever casting section can be predicted according to Eq. 24, and then the elevation prediction sequence can be formed by reduction generation (accumulation). The four previous predictions are compared with the data series shown in Eq. 20 to determine whether error correction needs to be performed.

## Elevation monitoring in bridge construction

**Fig 4:**
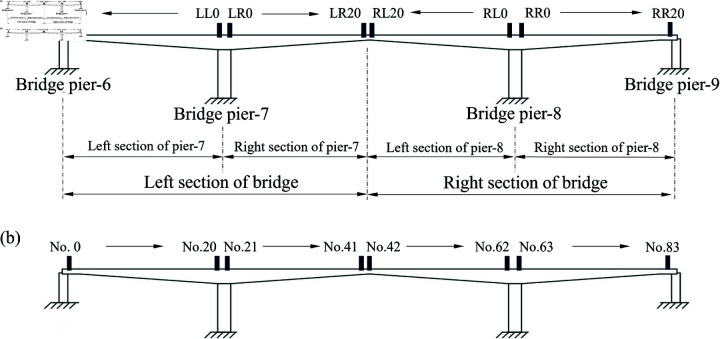
Specification management of cantilevers during bride construction. (**a**) Naming and (**b**) numbering of all construction cantilever sections.

Taking the construction of a bridge in Shanxi as an example, each cantilever section of the main girder is firstly distinguished for subsequent analysis. As shown in Fig. 4a, the bridge has two main piers (piers-7 and 8) and two side piers (piers-6 and 9). The midpoint of mid-span joint is marked as a distinction, the side near pier-7 is the left section of bridge, and the section near pier-8 is the right section of bridge. Furthermore, the two main piers also serve as demarcation points, and they are flanked by the left and right sections of either pier-7 or 8, the four main girders contain 20 cantilevers that need to be poured in sequence. Therefore, the direction and cantilever number are used to name the cantilever for each construction phase, e.g., LL20 denotes the cantilever section-20 in bridge’s left section, which is also located at the left section of pier-7. Additionally, the length of each cantilever is generally consistent, but the cantilever on either side of the abutment is determined based on the actual construction conditions. Furthermore, as shown in Fig. 4b all constructed cantilever sections of the bridge from left to right are numbered from No. 1 to No. 83, which facilitates subsequent analysis.

**Fig 5:**
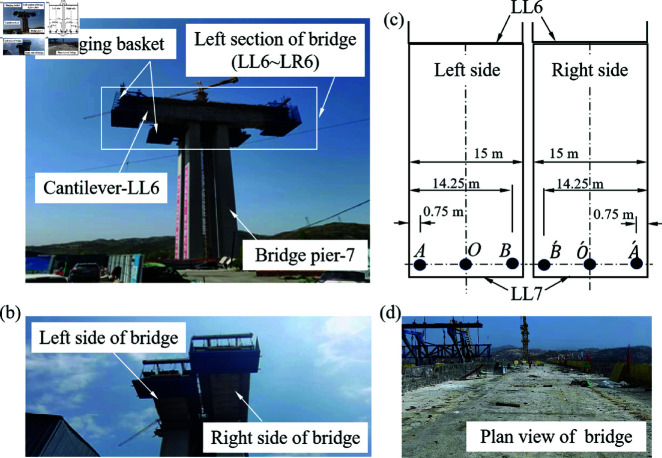
Photographs of construction bridges and locations of monitoring points. (**a**) Construction of cantilever section for main girder around bridge pier-7, (**b**) left and right sides of the bridge, (**c**) location of elevation monitoring for construction of the cantilever section and (**d**) a plan view of bridge.

In actual bridge construction, the cantilever 0 on the main pier is poured firstly, and then baskets are hung to each end and cantilevers 1 to 20 are poured in turn at intervals, as shown in Figs. 4a and 5a. Before the construction of a cantilever, deflection or elevation observations of the previous cantilever to which it is connected are required to ensure the linearity and stability for the final completed bridge. Therefore, the monitoring position of each cantilever should be set at the end of last cantilever section, as shown in Fig. 5c. Three elevation monitoring points are installed in the currently constructed cantilever, one of which is in the middle of cantilever end (*O* and $O^{\prime }$), and the other two are symmetrically distributed along the main girder (*A*, *B* and $A^{\prime }$, $B^{\prime }$), with a distance of 0.75 m from both sides, which not only measures the cantilever’s deflection, it can also observes the torsional deformation. Notably, the bridge has left and right sides, and the same observation method should be implemented on both sides, as shown in Fig. 5b. A plan view of the bridge during construction in Fig. 5d.

The elevation is obtained by measuring the spatial coordinates and deformation values of the construction cantilever using a total station (TCR1201+R400) and a level meter (DSZ01), respectively. Further, since human monitoring of elevation also generates errors, and in order to minimize such errors caused by human operations, the final elevation is derived by averaging ten measurements. On the other hand, temperature variations, especially daytime temperature differences, have a particularly significant effect on the deflection of main girder during construction. To minimize the effect of temperature, the deflection monitoring is scheduled in the morning before the sun rises. After obtaining the actual measured elevation, the actual measured value is compared with the predicted value, and if the error between the two is within a reasonable range, the construction of the next cantilever can be continued according to the prediction model. When the error is overlarge, the prediction model needs to be corrected until the corrected error meets the requirements.

## Analysis of bridge construction control

The most basic requirement for bridge construction control is to ensure the structure’s safety during construction, and it is necessary to keep the structure’s linearity and internal force in accordance with design requirements. For the bridge linear control, the measured deflection during the construction period is compared with the theoretical design deflection, and error analysis and correction prediction should be carried out to obtain the appropriate construction elevation of next cantilever section. Therefore, the results of linearity control after bridge completion and the control data during construction are analyzed, which come to check the conformity of results for bridge construction control.

### Linearity control result

**Fig 6:**
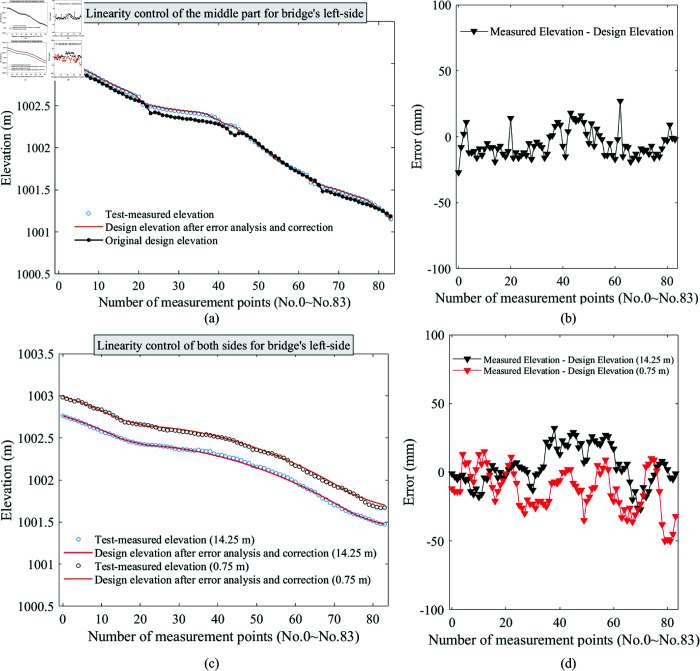
Elevations and linearities of the left-side bridge on the (a) center and (c) both-sides, which include elevations obtained from actual measurements and error-corrected design elevations. Elevation errors at (a) center and (c) both-sides of left-side bridge.

The completed bridge linearity is first analyzed, and data are collected from 84 monitoring sections from bridge’s left section to the right. The linearity of the center and both-sides for left-side bridge are shown in Fig. 6a and 6c. Owing to the bridge’s geographic location and engineering requirements, the elevation decreases continuously along the direction of increasing observation section number. Additionally, the elevations of the measured cantilever sections are basically consistent with the design elevations after error correction, with an error of less than 30 mm, as shown in Fig. 6b and 6d, where the error is calculated by subtracting the measured elevation from design elevation. Moreover, the linear trends measured in the middle and both sides of left-side bridge are in agreement, which further illustrates the effectiveness of structural deformation control in bridge construction control based on error analysis.

**Fig 7:**
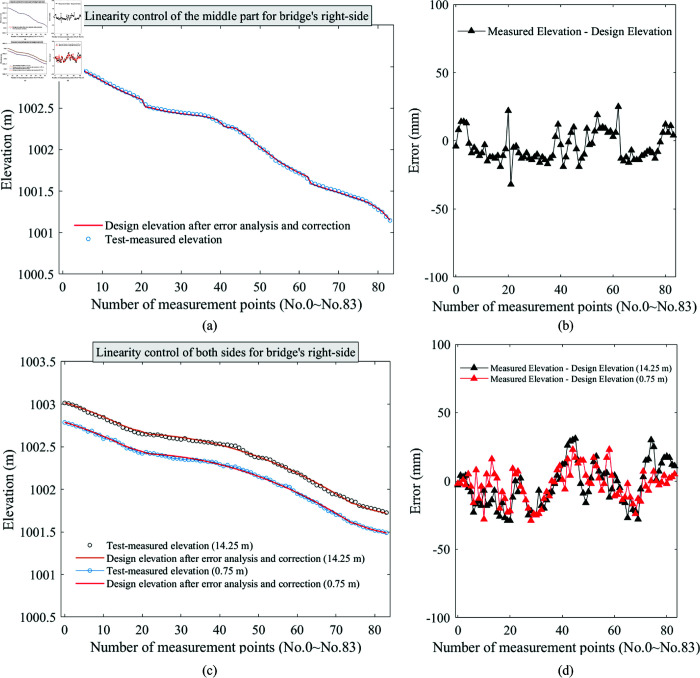
Elevations and linearities of the right-side bridge on the (a) center and (c) both-sides, which include elevations obtained from actual measurements and error-corrected design elevations. Elevation errors at (a) center and (c) both-sides of right-side bridge.

Similarly, the linearities of bridge’s right-side shown in Fig. 5b are analyzed in Fig. 7a and 7c, which plots the elevations of bridge’s right-side located at the center and both-sides after the completion of bridge construction, which are obtained from measurements and design with error corrections. Consistent with the left-side of bridge, the measured elevations along the entire bridge on right-side are basically same with the theoretical elevations obtained after error modification. And all errors are controlled within 30 mm, which is given in Fig. 7b and 7d. However, the elevation at right-side at the bridge’s center is slightly higher than that on left-side, which is determined by the actual engineering requirements.

In this study, error analysis is applied to obtain the optimal elevation of bridge’s each cantilever construction. The elevation of current construction cantilever is measured and compared with the original design value, and the reason for error between the two is evaluated, and the original design value is revised to correct the error before proceeding to construction of next cantilever. This can ensure the linearity and strength of when bridge construction completes. Obviously, it has been preliminarily confirmed the role of error analysis in bridge construction control from bridge linearities in Figs. 6 and 7.

### Elevation control for cantilever construction

In the implementation of construction control for a bridge in Shanxi, the construction process of each cantilever is divided into four phases, namely: basket positioning, concrete pouring, tensioned prestressing steel, and forward movement of basket. There is a necessity to focus on elevation control of the cantilever after construction and tensioning prestress, respectively. The elevation control data during the construction of main girder from section LL0  ∼  LL20 are randomly selected for analysis.

**Fig 8:**
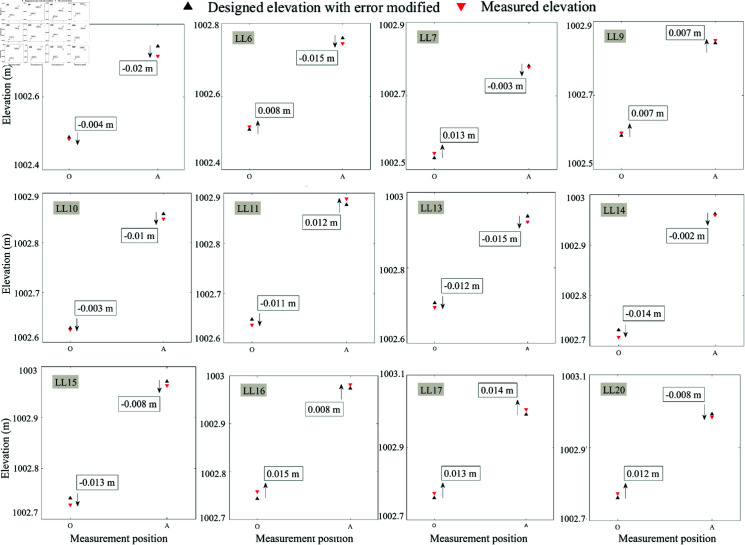
Measured and theoretical elevations with error corrected of cantilevers LL5 ∼ LL20 after pouring during the construction of the left-side bridge.

Fig. 8 plots the measured elevation and the corrected design elevation with error analysis derived from the last constructed cantilever section during the pouring phase, and they are obtained from cantilever LL0  ∼  LL20 in bridge’s left-section. According to the gray system theoretical model in Section 2.4, the cantilever construction of LL1  ∼  LL4 should be carried out empirically, while the cantilevers from LL5 to LL20 are built by using the theoretical design with error correction mentioned in Section 2. As can be seen from Fig. 8, the error between the theoretical and measured values generated in the center of bridge’s left-section (O) during the construction for each section is not more than 15 mm, and the error between the two generated on one-sides of the bridge’s left-section (A) is less than 20 mm, which are smaller than the errors when the bridge is completed, as shown in Fig. 6. This phenomenon is probably explained by the fact that the construction of subsequent cantilever had an unnegligible effect on the deflection on current cantilever.

**Fig 9:**
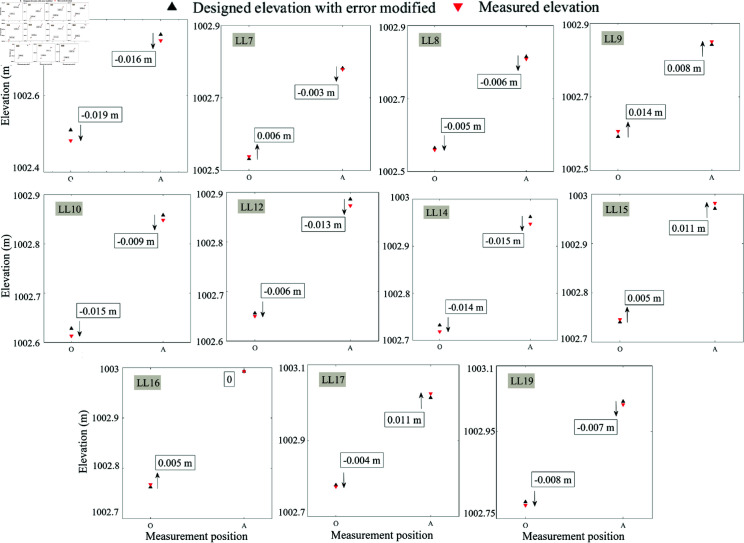
Measured and theoretical elevations with error corrected of cantilevers LL5 ∼ LL20 after tensioning prestressing steel during the construction of the left-side bridge.

Then, the measured and modified design values of elevation in the center (O) and one-side (A) of cantilever after tensioning prestressing steel are also listed. As shown in Fig. 9, most constructional data of cantilevers in LL5  ∼  LL20 are shown by engineering protocols. It can be noticed that when the cantilever was tensioned with prestressing steel, there is a slight effect on its deflection and elevation, however, this stage reduces the error between measured and measured elevation overall.

Since the bridge construction is critical and irreversible, to validate the effectiveness of proposed method for error analysis and correction in bridge construction control, it can only be achieved by analyzing the linearity of completed bridge and the difference between the designed elevation and measured one in every construction stage. By analyzing Figs. 6–Fig. 9, it can be inferred that at the beginning of one cantilever’s construction, by comparing the measured elevation of previous cantilever with the original design elevation, and further correcting the difference between the two, the revised design elevation can be obtained eventually. The proposed method can make the error generated during bridge cantilever’s construction less than 20 mm, and keep the error of bridge’s elevation after finishing the bridge’s construction less than 30 mm.

## Conclusions

This paper proposes a method for controlling structural deformation in bridge construction control based on error analysis, which is aimed at minimizing the influence of process, material, temperature and environmental factors on the theoretical design, thereby the reasonable linearity and structural strength of the bridge can be guaranteed. The main conclusions is shown as follows:

1. The theoretical elevation is obtained by establishing the deflection model of constructed cantilever, and the effect of subsequent cantilevers on the deflection in current stage is considered. And the errors in elevation caused by temperature and time interval are further calculated. Finally, the linear minimum variance estimation is introduced to correct the errors.
2. A practical application is carried out in the construction of a bridge in Shanxi Province, where the proposed error analysis method is further implemented by measuring the current cantilever elevation and comparing it with the original design value, with the aim of obtaining a reasonable elevation for the next cantilever. The results show that, after introducing the error analysis and correction, the elevation error generated during the construction is less than 20 mm, and the elevation error after the bridge is completed is less than 30 mm.

Significantly, the error analysis mainly relies on the actual measurement data, and in the actual bridge construction process, the measurement data are always affected by a variety of factors, such as the measurement device’s accuracy, tester’s operation, and environment, which leads to a certain degree of discrepancy between it and the actual situation. Therefore, future research will focus on improving the measurement accuracy of bridge engineering.

## Supporting information

S8 and S9 Figs Measured and theoretical elevations with error corrected of cantilevers LL5 ∼ LL20 after pouring and tensioning prestressing steel during the construction of the left-side bridge.
pptx
