# Supplementary material for: Structural deformation control in bridge construction based on error analysis and correction
Source: PLoS One. 2025 Apr 14;20(4):e0319844. doi: 10.1371/journal.pone.0319844 (PMC11996214; doi:10.1371/journal.pone.0319844)
Supplement: S8 and S9 Figs — Measured and theoretical elevations with error corrected of cantilevers LL5 ∼ LL20 after pouring and tensioning prestressing steel during the construction of the left-side bridge. pptx [file pone.0319844.s001.pptx]

## Slide 1
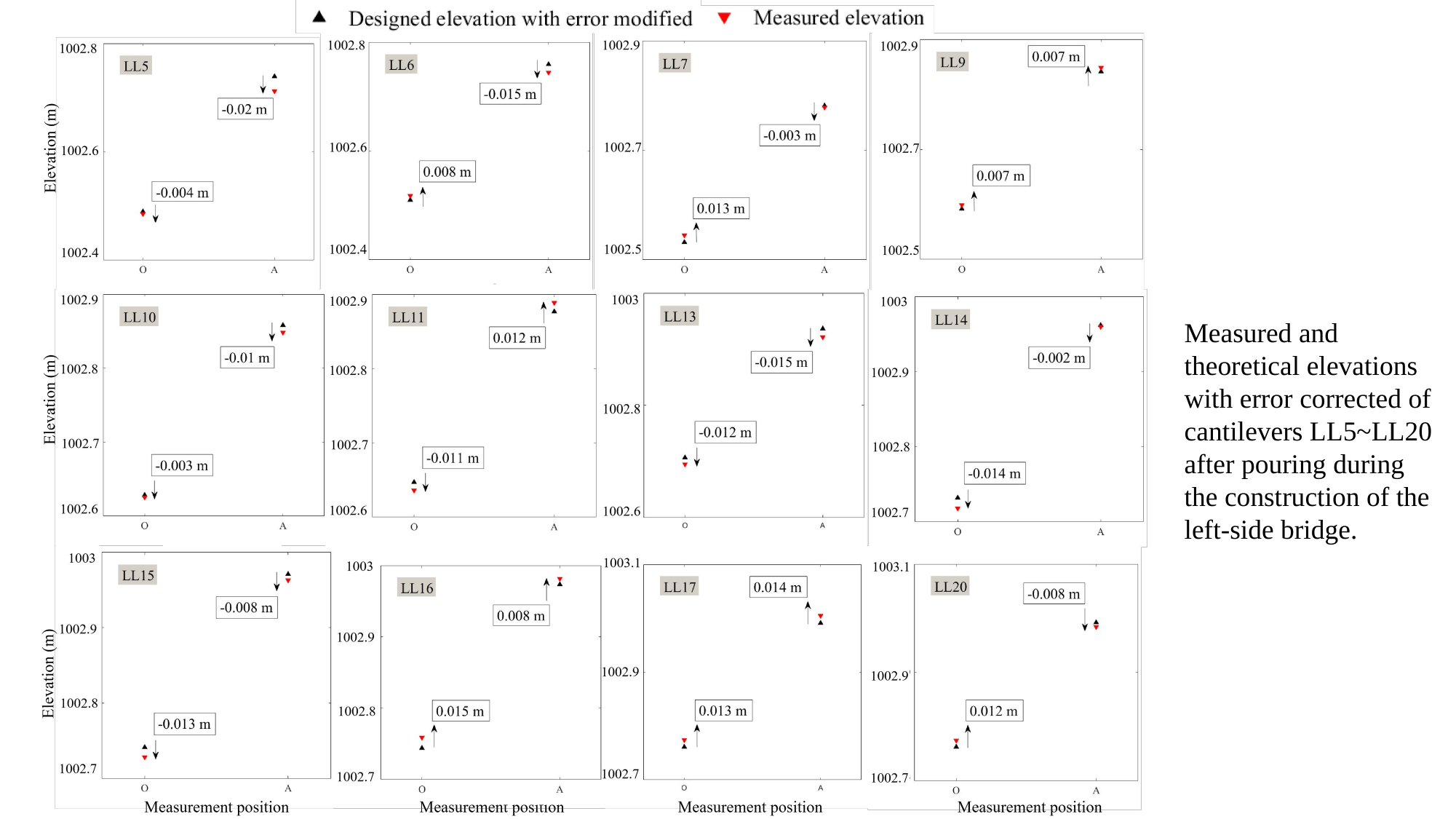

Measured and theoretical elevations with error corrected of cantilevers LL5~LL20 after pouring during the construction of the left-side bridge.

## Slide 2
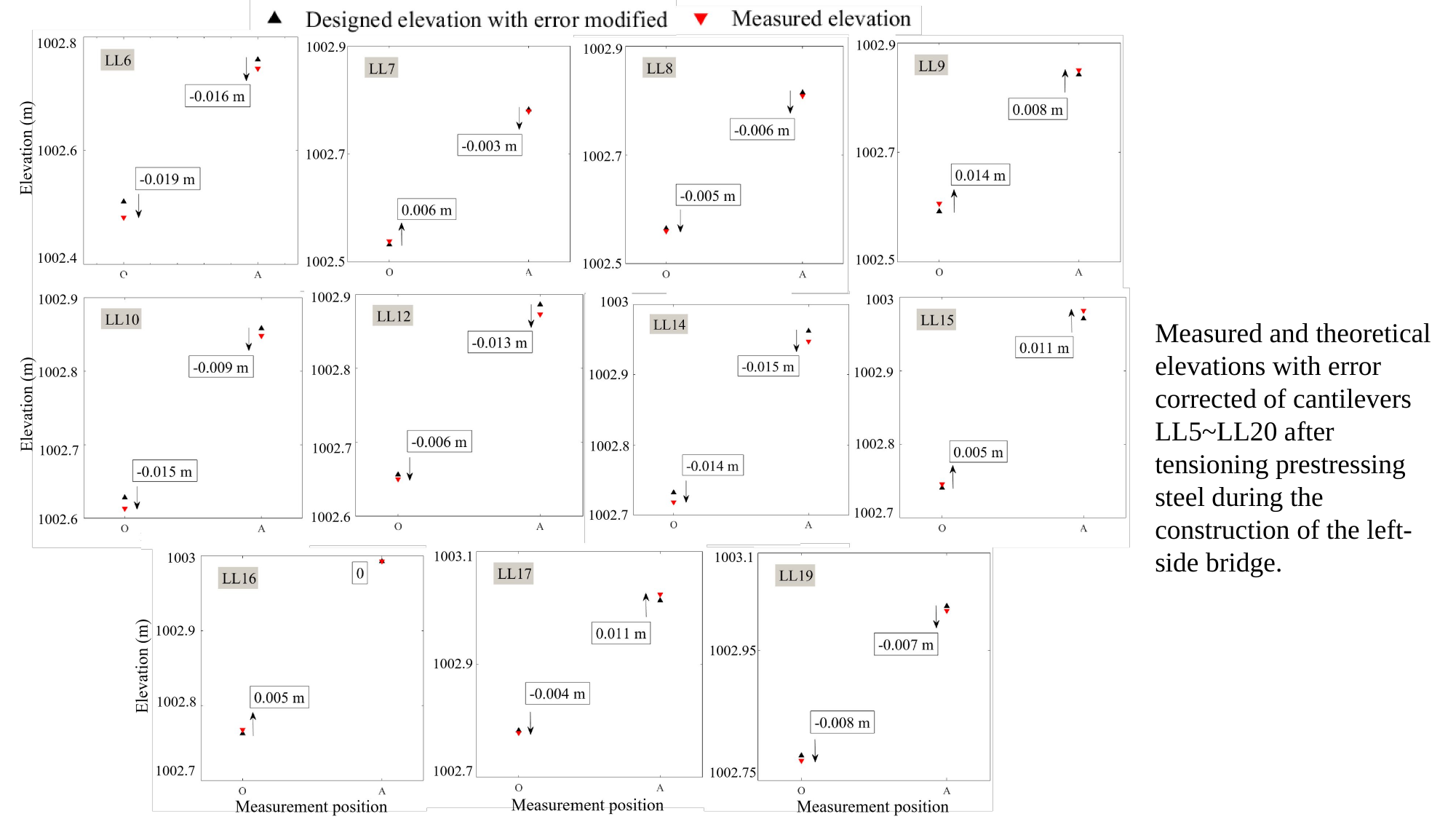

Measured and theoretical elevations with error corrected of cantilevers LL5~LL20 after tensioning prestressing steel during the construction of the left-side bridge.
